# 2023–2024: Selected Papers from the ‘Polymer Chemistry’ Section

**DOI:** 10.3390/polym17202768

**Published:** 2025-10-16

**Authors:** Shin-ichi Yusa

**Affiliations:** Department of Applied Chemistry, Graduate School of Engineering, University of Hyogo, Shosha 2167, Himeji 671-2280, Hyogo, Japan; yusa@eng.u-hyogo.ac.jp; Tel.: +81-79-267-4954

In recent years, the design, processing, and functional performance of materials have become increasingly integrated. Against this backdrop, polymer chemistry research has accelerated, with a dual focus on “sustainability” and “practical implementation”. This Editorial offers a broad overview of the interface between fundamental research and applications, including multicomponent membranes formed via non-equilibrium self-assembly, a state-of-the-art review of anion exchange membranes for green water electrolysis, and advances in long-wavelength-sensitive cationic photoinitiating systems. Within, I briefly summarize the originality and technical significance of each paper and outline future challenges, including the standardization of evaluation methods, and quantitative frameworks for environmental compatibility.

“Non-Equilibrium Block Copolymer Self-Assembly Based Porous Membrane Formation Processes Employing Multicomponent Systems” [1] reviews design principles for hierarchically porous membranes formed by combining block copolymer self-assembly with self-assembly and non-solvent-induced phase separation (SNIPS). The article shows that an ordered top separation layer supported by an asymmetric hierarchical substructure can ease the usual trade-off between selectivity and permeability, demonstrating how solvent composition, additives, and evaporation conditions steer the evolution of the surface structure, drawing on scattering analyses. It also explains that multicomponent casting solutions containing small molecules, inorganic precursors, or nanoparticles allow the precise tuning of pore size and chemistry and even conversion to carbon, metals, or nitrides. Recent research directions are covered as well, including pore size control by co-assembly with non-solvent-induced phase separation (CNIPS) using glycerol and surface SNIPS (S2NIPS), which yields a dual-layer membrane with a block copolymer-based mesoporous separation layer on a homopolymer support. Overall, the review outlines a versatile platform for future membrane technologies and foundational materials science.

“Recent Advancements of Polymeric Membranes in Anion Exchange Membrane Water Electrolyzer (AEMWE): A Critical Review” [2] outlines the state of polymer design, durability, and cell integration for anion exchange membranes used in alkaline water electrolysis. This review examines the alkaline stability of cationic sites such as quaternized polyphenylene, imidazolium, and piperidinium, the resistance of polymer backbones to hydrolysis and Hofmann elimination, and the effects of crosslinking and blending. The review also covers cell-engineering issues, including gas-diffusion electrodes, catalyst selection, carbonate precipitation control, and humidity and temperature management during scale-up, and it identifies conditions that balance performance (conductivity, mechanical properties, tolerance to feed-water impurities) with durability. In addition, it outlines methods of lowering system costs by pairing pure-water operation with low-Pt or platinum group metal (PGM)-lean catalysts, and calls for harmonized standards and testing protocols to support implementation.

“Recent Advances and Challenges in Long Wavelength Sensitive Cationic Photoinitiating Systems” [3] surveys cationic photoinitiating systems that operate at longer wavelengths and organizes design strategies compatible with ultraviolet (UV) and visible light-emitting diodes (LEDs). The review frames two routes to red shift activation: single-component photoinitiators with extended conjugation or chromophore linkage, and multicomponent systems that use photosensitization through energy or electron transfer. It covers iodonium and sulfonium salts; ferrocenium, phosphonium, ammonium and nonionic photoacid generators; and charge transfer complexes, exciplex sensitization and free radical-promoted cationic polymerization. These approaches enable curing under blue and green LEDs and, with upconversion, even under near-infrared (NIR) light, which broadens applicability to thick or highly scattering samples and to digital light processing (DLP)-based 3D printing. Overall, the article provides practical guidance for combining materials design with optical design while leveraging the intrinsic advantages of cationic photopolymerization, including oxygen tolerance, low shrinkage and the possibility of dark curing.

“Lignocellulose Biomass Liquefaction: Process and Applications Development as Polyurethane Foams” [4] reviews methods of converting liquefied lignocellulose into polyols and then into polyurethane foams, including feedstock pretreatment, acid catalysts, solvent systems, and operating conditions. It summarizes how functionality distribution, average molecular weight, and residual solids in bio-based polyols affect foaming reactions, cell morphology, and mechanical performance, and it shows that property tuning is possible through blending with petrochemical polyols and catalyst selection. The article compiles property data for building applications such as thermal insulation and sound absorption and extends the discussion to life-cycle and cost assessments that frame these materials as sustainable options. It also contrasts moderate acid-catalyzed solvolysis in polyols with hydrothermal liquefaction and outlines process intensification using microwave or ultrasound. Practical bottlenecks are identified, including byproduct and residue management, scale-ups from batch to continuous operation, and the need for specifications and standardized testing, leading to a roadmap for greener polyurethane implementation.

“Recycled PLA for 3D Printing: A Comparison of Recycled PLA Filaments from Waste of Different Origins after Repeated Cycles of Extrusion” [5] is a demonstration of repeated re-extrusion of two types of poly(lactic acid) (PLA) waste, namely a single known commercial grade and a mixed stream derived from personal protective equipment, into filaments without additives. The study compares degradation across cycles using diffusion-ordered spectroscopy (DOSY) NMR, solution viscometry, Fourier transform Infrared (FTIR) spectroscopy, and mechanical testing. The known grade shows a slower decline in molecular weight and retains practical properties for about three re-extrusion cycles, whereas the mixed stream reaches its limit after about two cycles. FTIR indicates no significant contamination from other polymers, and the initial molecular weight emerges as a key factor governing recyclability. The results also underscore the importance of controlled washing, drying, and extrusion conditions for reproducible outcomes. In addition, the authors propose collection and mechanical recycling models for distributed settings in universities and communities, with scenarios that assume full or limited traceability.

“Nanocrystalline Cellulose-Supported Iron Oxide Composite Materials for High-Performance Lithium-Ion Batteries” [6] reports an electrode in which nanocrystalline cellulose (NCC) serves as both a template and a conductive carbon source to anchor nanosized Fe_2_O_3_, yielding an NCC-Fe_2_O_3_ composite by simple thermal treatment. The negative surface charge, high surface area, and rigidity of NCC suppress particle aggregation and buffer volume change, which leads to high capacity retention during long cycling. The electrode delivers an initial capacity near 1000 mAh g^−1^ and maintains about 577 mAh g^−1^ after 1000 cycles at 100 mA g^−1^, while also showing strong rate capability. Microscopy and spectroscopy confirm homogeneous Fe_2_O_3_ dispersion on a meso- to macroporous carbon network that facilitates electron and ion transport. The study also discusses how surface carboxyl and sodium carboxylate groups on NCC assist stable solid-electrolyte interphase (SEI) formation. Overall, the synergy between a bio-based carbon framework and iron oxide enables durable, high-energy lithium storage.

“Effects of Ultrasonication in Water and Isopropyl Alcohol on High-Crystalline Cellulose: A FTIR and X-ray Diffraction Investigation” [7] is an experimental study that quantitatively evaluates how ultrasonication in water and in isopropyl alcohol affects high-crystalline cellulose using FTIR and X-ray diffraction (XRD). By systematically varying sonication time, output amplitude, liquid medium, and vessel geometry, the authors analyze changes in crystallinity indices and in hydrogen-bonding energy and distance. In isopropyl alcohol, crystallinity tends to increase under suitable conditions, suggesting improved dispersion with limited structural damage. In water, prolonged sonication reduces the crystalline region, indicating that differences in solvent properties influence cavitation behavior and the stability of the crystalline phase. Differences in vessel geometry and amplitude also produce significant changes in the crystallinity indices, clarifying the links between process parameters and structural responses.

“In Situ Monitoring of the Curing of Highly Filled Epoxy Molding Compounds: The Influence of Reaction Type and Silica Content on Cure Kinetic Models” [8] monitors the curing of silica-filled epoxies in situ by dielectric analysis (DEA) and benchmarks the online data against offline differential scanning calorimetry (DSC) to build model-free kinetics using the Friedman approach. Resin systems with different reaction rates are compared under processing conditions below and above the final glass-transition temperature (Tg), and silica loadings of about 73 to 83 wt% are shown to affect reaction rate, apparent activation energy, and sensor sensitivity. DEA tracks gelation and chemical shrinkage continuously through the ion viscosity signal, and together with DSC enables construction of time temperature transformation (TTT) diagrams that visualize Tg progression and the onset of diffusion control via the DiBenedetto equation. The outcome is a predictive framework that supports process window definition, reduction in residual stress, and quality assurance, demonstrating that lowering silica content accelerates curing while improving signal-to-noise.

“Multi-Material 3D Printing of Biobased Epoxy Resins” [9] presents a dual-vat digital light processing (DLP) platform that prints multi-material parts from biobased epoxies, specifically epoxidized linseed oil (ELO) and vanillin alcohol diglycidyl ether (DGEVA). The authors optimize photocuring by pairing an iodonium photoinitiator with ITX as a photosensitizer and Sudan II as a photo-absorber, and they follow reactivity and conversion with photo-DSC and FTIR. Jacob’s working curves are used to set per-layer exposure, and an automated vat-switching and sponge-cleaning step limits cross-contamination of uncured resin. By combining a lower-Tg, lower-stiffness ELO network with a higher-Tg, stiffer DGEVA network in a single build, the study shows synergistic behavior: DMTA indicates that multilayer Tg shifts toward the DGEVA value. Tensile and flexural tests place the multi-material specimens between the single-material extremes, allowing stiffness, strength, and elongation to be tuned by layer sequence and fraction. A flower-shaped specimen demonstrates temperature-activated shape memory, highlighting a practical route to sustainable multi-material epoxy printing.

“Redox-Stable and Multicolor Electrochromic Polyamides with Four Triarylamine Cores in the Repeating Unit” [10] reports aromatic polyamides that incorporate four triarylamine (TPA) cores within each repeating unit and demonstrate multicolor electrochromism with excellent redox stability. Leveraging the electron-donating character of methoxy substituents and resonance stabilization afforded by naphthyl units, the materials exhibit durability that surpasses previously reported TPA systems. For example, the decline in coloration efficiency upon the first oxidation remains within only a few percent even after 14,000 cycles. Together with high contrast across the visible-to-NIR region, fast response, and solution processability, these features point to strong potential for smart windows and NIR light management devices. The effectiveness of the molecular design, namely the multicore architecture and strategic placement of electron-donating groups, is substantiated by electrochemical, spectroscopic, and thin film device evaluations.

The ten papers highlighted in this Editorial demonstrate how polymer chemistry can advance both sustainability and practical implementation by integrating molecular design, hierarchical structure formation, process optimization, and device-level validation. I hope these concrete examples will further promote academia–industry collaboration and serve as a springboard for accelerating the deployment of standardized, scalable, and environmentally responsible next-generation polymer technologies. I also look forward to the ‘Polymer Chemistry’ Section’s continued growth as a vibrant venue for rigorous and impactful research.
